# A locus-specific database for mutations in *GDAP1 *allows analysis of genotype-phenotype correlations in Charcot-Marie-Tooth diseases type 4A and 2K

**DOI:** 10.1186/1750-1172-6-87

**Published:** 2011-12-26

**Authors:** Julien Cassereau, Arnaud Chevrollier, Dominique Bonneau, Christophe Verny, Vincent Procaccio, Pascal Reynier, Marc Ferré

**Affiliations:** 1 INSERM, U771, Angers, F-49000, France; 2 CNRS, 6214, F-49000 Angers, France; 3 Univ Angers, Angers, F-49000, France; 4 CHU Angers, Département de Neurologie, Angers, F-49000, France; 5 CHU Angers, Département de Biochimie et Génétique, Angers, F-49000, France

**Keywords:** inherited peripheral neuropathy, Charcot-Marie-Tooth disease (CMT), ganglioside-induced differentiation-associated protein 1 (GDAP1), locus-specific database (LSDB)

## Abstract

**Background:**

The ganglioside-induced differentiation-associated protein 1 gene (*GDAP1*), which is involved in the Charcot-Marie-Tooth disease (CMT), the most commonly inherited peripheral neuropathy, encodes a protein anchored to the mitochondrial outer membrane. The phenotypic presentations of patients carrying *GDAP1 *mutations are heterogeneous, making it difficult to determine genotype-phenotype correlations, since the majority of the mutations have been found in only a few unrelated patients. Locus-specific databases (LSDB) established in the framework of the Human Variome Project provide powerful tools for the investigation of such rare diseases.

**Methods and Results:**

We report the development of a publicly accessible LSDB for the *GDAP1 *gene. The *GDAP1* LSDB has adopted the Leiden Open-source Variation Database (LOVD) software platform. This database, which now contains 57 unique variants reported in 179 cases of CMT, offers a detailed description of the molecular, clinical and electrophysiological data of the patients. The usefulness of the *GDAP1 *database is illustrated by the finding that *GDAP1 *mutations lead to primary axonal damage in CMT, with secondary demyelination in the more severe cases of the disease.

**Conclusion:**

Findings of this nature should lead to a better understanding of the pathophysiology of CMT. Finally, the *GDAP1 *LSDB, which is part of the mitodyn.org portal of databases of genes incriminated in disorders involving mitochondrial dynamics and bioenergetics, should yield new insights into mitochondrial diseases.

## Background

Charcot-Marie-Tooth (CMT) disease is one of the commonest inherited peripheral neuropathies with a prevalence of about 10-30 per 100,000, depending on the country of origin [1]. CMT neuropathies are characterized clinically by progressive sensory loss, weakness, muscle atrophy, loss of deep tendon reflexes, and foot deformities. To date, about forty genes have been incriminated in CMT. Some of these genes encode mitochondrial proteins, i.e. mitofusin 2 (*MFN2*) [MIM:608507] and the ganglioside-induced differentiation-associated-protein 1 (*GDAP1*) [MIM:606598]. *GDAP1 *mutations are responsible: for CMT4A [MIM:214400] [2], the most frequent recessive subtype of demyelinating CMT; for AR-CMT2 [MIM:607706] [3], the axonal recessive subtype; for CMTRIA [MIM:608340] [4], the intermediate recessive CMT; and for CMT2K [MIM:607831] [5-7], the rare dominant subtype.

The gene locus, first identified in cases of CMT4A in Tunisian families on chromosome 8q13-q21 (CMT4A), is associated with slow motor nerve conduction velocity, and hypomyelination as detected by nerve biopsy [8]. The corresponding gene, *GDAP1*, was identified by Baxter et al. (2002). *GDAP1 *was previously identified as one of ten cDNAs expressed in a differentiated Neuro2a mouse neuroblastoma cell line, in which cholinergic differentiation with neurite sprouting had been induced by transfection of GD3 synthase (a2,8 sialyltransferase) cDNA [9].

The *GDAP1 *gene spans ~17 kilobases of genomic DNA, containing six exons and five introns. Two transcript variants of *GDAP1 *have been identified. Transcript variant 1, representing the longer variant, encodes the longer isoform, designated as isoform a (358 amino acids). Transcript variant 2, encoding isoform b, contains an alternate in-frame exon, uses an alternate splice site in the 5'-coding region and a downstream start codon, compared to transcript variant 1. Isoform b (290 amino acids) has a shorter N-terminal sequence compared to isoform a. mRNA studies using RT-PCR have demonstrated the ubiquitous expression of *GDAP1*, predominantly in nervous tissues [3].

Phylogenetic and structural analyses suggest that GDAP1 belongs to a subfamily of glutathione-S-transferases (GSTs) [3,10,11]. GDAP1 has two typical GST domains, domain I (GST-N) at the N-terminal region and domain II (GST-C) at the C-terminal region. The existence of these domains was predicted by using bioinformatics tools [3,10] but no functional GST activity has been demonstrated so far [11,12]. It has also been shown that GDAP1 has a single transmembrane domain (TMD) at the extremity of the C-terminal, and a hydrophobic domain (HD) in the flanking C-terminal region [10,13]. Moreover, GDAP1 has two additional regions between amino acids 152-164 and 169-195 that are predicted to represent two helices, α4 and α5, constituting the α4-α5 loop.

Several studies have suggested that GDAP1 plays a role in mitochondrial fission [14,15]. It was shown that the TMD plays a key role in mitochondrial targeting and mitochondrial membrane insertion [13]. Positively charged amino acids flanking the TMD at the C-terminal are involved in mitochondrial targeting and the fission function of GDAP1. The HD seems essential for the mitochondrial fission mediated by GDAP1. We have recently shown that GDAP1 mutations in fibroblasts from CMT2K patients are associated with defective mitochondrial complex I activity [5].

The examination of the relationship between the genotype and the phenotype of the various forms of CMT disease involving *GDAP1 *mutations shows that the recessive forms are generally far more severe than the dominant forms. In particular, the truncating mutations have a more severe phenotype in the recessive forms with early onset and rapid progression of the disease leading to the inability to walk by the age of 10, generally followed by wheelchair-dependence before the end of the third decade [16].

However, the phenotypic presentations of patients carrying *GDAP1 *mutations are heterogeneous. There is not only a large clinical variability concerning the ages of onset and functional disability among patients carrying the same mutations [17] but also a significant intra-familial variability [18]. Since the majority of mutations have been described in only a few unrelated patients, it is difficult to determine phenotype-genotype correlations for *GDAP1 *gene mutations. The analysis of a larger number of cases of CMT involving *GDAP1 *mutations, along with detailed clinical, electrophysiological and pathological data, will be required to establish a reliable relationship between the genotype and phenotype of the different forms of the disease.

The increasing importance of *GDAP1 *in CMT prompted us to develop a web-based locus-specific database (LSDB) for *GDAP1 *sequence variations with short publication delays. To our knowledge, the only other database collecting *GDAP1 *mutations is the Inherited Peripheral Neuropathies Mutation Database (IPNMDB) [19], which includes 29 *GDAP1 *mutations among the 953 mutations in 44 genes related to inherited peripheral neuropathies. Our LSDB aims at collecting all the *GDAP1 *mutations incriminated in disorders involving mitochondrial dynamics and bioenergetics, with a full record of clinical, electrophysiological and biochemical data. We here describe the construction of the database, the procedure for data submission, and the presentation of the data.

## Methods

**Figure 1:**
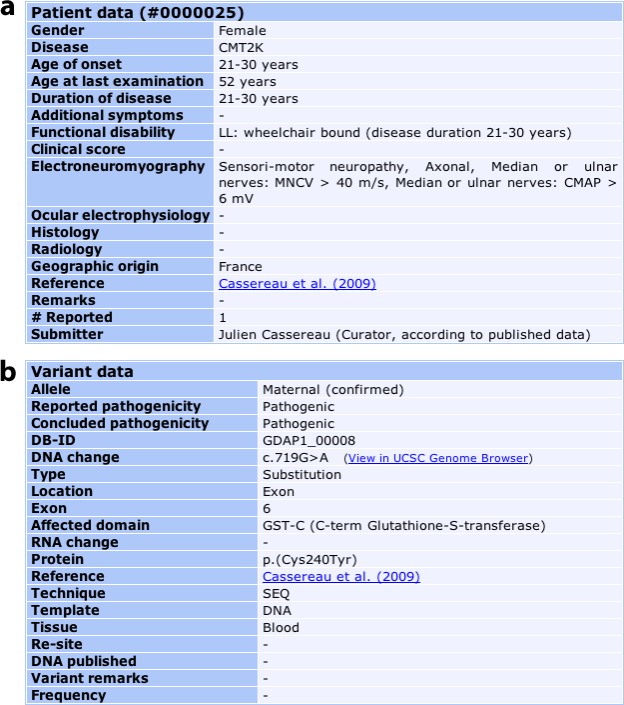
**Sample record in the *GDAP1 *database. a: **clinical items; and **b: **molecular items. MNCV: Motor Nervous Conduction Velocity (in m/s); CMAP: Compound Motor Action Potential (in mV); LL: lower limbs.

We have established a locus-specific database for *GDAP1 *using the Leiden Open-source Variation Database (LOVD) version 2 build 31 system [20]. This database includes a total of 20 variant items and 18 clinical patient items. Figure 1 shows a typical entry. A standardized description of the clinical and molecular items is set up using drop-down lists with predefined variables. The clinical features are based on the usual symptoms of mitochondrial diseases.

The data is openly accessible and should prove a valuable tool for clinicians and researchers alike since it will contain published as well as unpublished sequence variants. Contributors can submit their variants online to the database after registering for a login and password. This contact information is collected for reference purposes and clarification of the data submitted. The description of mutational data is confirmed using the web-based software Mutalyzer, to which the LOVD software is currently also linked [21]. Variations submitted directly to the LSDB are re-checked with these tools.

The *GDAP1 *database reviews all the clinical and molecular data of patients carrying *GDAP1 *mutations published in peer-reviewed literature, as well as unpublished contributions that may be directly submitted. While most variants can be described in terms of the latest update of the standard nomenclature [22,23], some inaccuracies may persist because gene anomalies discovered earlier might have been named according to a convention now out of use. Eventually, the *DNA published *field of the page dedicated to each variant (Figure 1b) indicates whether the published name of the mutation has been modified by the curator. The *GDAP1 *LSDB website requires absolute compliance with the rules set out below to describe sequence variants in order to provide uniform and comparable data.

The *GDAP1 *mutations are described according to the *GDAP1 *transcript variant 1 [RefSeq:NM_018972.2], containing 6 exons and encoding isoform a containing 358 amino acids. The nucleotide numbering of nuclear genes reflects cDNA numbering with +1 corresponding to the A of the ATG translation initiation codon in the reference sequence, according to the guidelines of the Human Genome Variation Society (HGVS). The initiation codon is codon 1.

The criteria of pathogenicity, dependent on the clinical context and molecular findings, are stated in the *Variant data *fields: *Reported pathogenicity *and *Concluded pathogenicity *(Figure 1b). Putative novel mutations detected in affected patients must segregate according to disease status and not be present in control individuals. Putative mutations are graded according to type of mutation: frameshift and nonsense mutations are considered to be pathogenic; missense mutations are described as being of *unknown pathogenicity *when detected in single families, or as *probably pathogenic *when detected in a group of families; the mutations are considered to be pathogenic when so proven by experimental evidence or detected in multiple families.

## Results and discussion

### Type of mutations and affected domains

**Figure 2:**
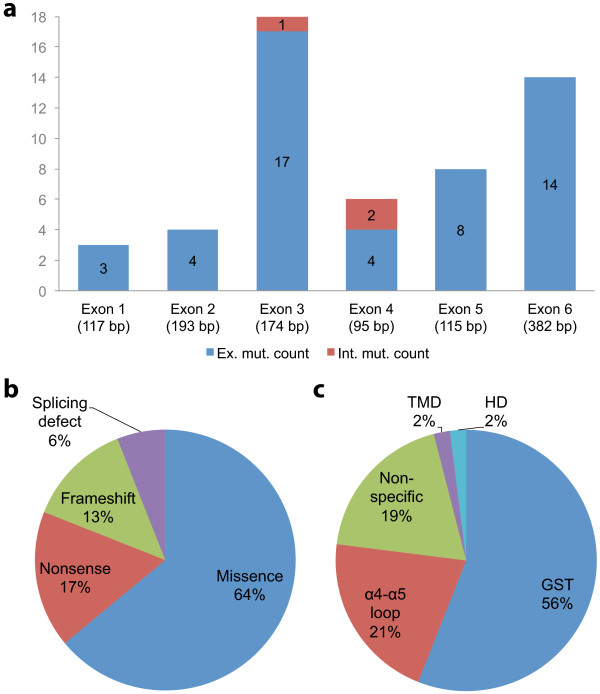
**Distribution of the 53 pathogenic mutations in the *GDAP1 *gene. a: **exons involved in the mutations are shown as blue bars; the mutations in the intronic neighbourhood of the exons are shown as red bars; **b: **type; and **c:** domain. bp: base pairs; GST: glutathione *S*-transferase; TMD: transmembrane domain; HD: hydrophobic domain.

To date, the *GDAP1 *database contains 57 unique variants, four of which are considered as non-pathogenic sequence variants (NPSV). Pathogenic mutations, which affect the coding sequence of the gene, are more frequently found in exon 3 (17 mutations) and exon 6 (14 mutations) (Figure 2a). The most frequent mutational protein consequences are missense mutations observed in 34 of the 53 variant mutations. In addition, nine frameshifts (18%), seven nonsense (14%) and three splicesite (6%) mutations have also been reported (Figure 2b).

Whereas few mutations are recurrent, some have been frequently reported: the c.581C>G mutation (p.Ser194X) reported 38 times in Spain, Morocco and Tunisia; the c.487C>T mutation (p.Gln163X) reported 34 times, mainly in Spain; the c.715C>T mutation (p.Leu239Phe) reported 28 times, mainly in Eastern Europe; and the c.358C>T mutation (p.Arg120Trp) 22 times, mainly in Spain.

Among all the pathogenic variants mentioned in the database, 56% are located in the glutathione *S*-transferase (GST) domains of the protein (Figure 2c), highlighting the important role of these domains in protein function, as recently reported [24]. Among the 34 missense mutations, 21 (62%) affect GST domains: 6 in GST-N and 15 in GST-C; 5 (14.5%) affect the α4-α5 loop; one affects the hydrophphobic domain, one affects the transmembrane domain; and 6 (17.5%) affect no particular domain of the protein. Interestingly, 5 of the 9 missense mutations involved in CMT2K are located in GST domains (p.Ser34Cys in GST-N, and p.Gln218Glu, p.Arg226Ser, p.Cys240Tyr and p.Pro274Leu in GST-C).

Similarly, 35 of the 45 patients (78%) presenting with the recessive form of the disease associated with homozygous missense mutations, harbor mutations located in GST domains (12 in GST-N and 23 in GST-C). In addition, truncating mutations located upstream of amino acid 287 affect at least one GST domain, as is the case in almost all truncating mutations reported.

### Clinical data

To date, the database includes 179 patients (93 females, 75 males and 11 not specified): 47 with a dominant form CMT2K, and 126 with a recessive form (CMT4A, AR-CMT2 or CMTRIA). Six patients were unclassified, either because the authors suspected mutations on the second allele, or because the pathogenicity of the second variant was uncertain. The database shows the clinical data for all the patients reported, with the exception of 28 patients for whom the data was unavailable or insufficient for classification of the phenotype.

Clinical severity was evaluated according to the age of onset of the disease, i.e. before age 10, between ages 11-30, and after age 30, and by walking ability, i.e. independent, requiring technical assistance with a cane or a crutch, or wheelchair-bound, before or after age 30. The phenotypic data correlated with the mutational data in patients with sufficient clinical data for each item:

- age of onset according to: (1) the mode of inheritance (dominant or recessive); and (2) the domains affected in the dominant forms of CMT (GST domains, α4-α5 loop, transmembrane domain, hydrophobic domain or non-specific domain); 

- disability in walking according to: (1) the mode of inheritance (dominant or recessive); and (2) the predicted effect at the protein level: truncating mutations or missense mutations.

Data analysis shows that patients with recessive forms of CMT, i.e. CMT4A and AR-CMT2, presented a more severe phenotype than those with the dominant form, i.e. CMT2K, with a more early onset and a more rapid evolution (Table 1). Interestingly, nineteen percent of patients with the dominant form of CMT were asymptomatic until age 30, whereas all patients with a recessive form had symptoms before the end of the adolescence. In addition, mutations affecting GST domains were responsible for a more severe phenotype probably produced by a different pathophysiological mechanism. Almost all patients with mutations in GST domains were symptomatic after age 50 whereas 17% of patients with mutations in non-specific domains were asymptomatic after the age of 50. These findings suggest that mutations affecting GST domains are responsible for a more severe phenotype probably due to a different pathophysiological mechanism.

**Table 1:** Age of onset of CMT

	Age of onset				Total
	< 10 y	10-30 y	> 30 y	asymptomatic > 50 y	
**Recessive forms**	120 (98%)	3 (2%)	0	0	123
**Dominant forms**	11 (25%)	18 (41%)	9 (20%)	6 (14%)	44
*Domain affected: *					
*GST*	*3 (33%)*	*2 (22%)*	*4 (44%)*	*0*	*9*
*Others*	*8 (23%)*	*16 (36%)*	*5 (14%)*	*6 (17%)*	*35*

Genotype-phenotype correlation analysis using *GDAP1 *locus-specific database data evaluating the age of onset according to mode of inheritance and affected domains in dominant forms. Values represent the number of patients for each situation; the corresponding percentages are shown in brackets. y: years; GST: glutathione *S*-transferase.

The course of CMT associated with *GDAP1 *mutations was more rapid in the recessive form than in the dominant forms (Table 2). All AR-CMT patients required technical support for walking after age 30. In contrast, among the CMT2K patients reported in the database, 78% walked independently after age 30. Nonsense and frameshift mutations were more often associated with a severe phenotype of CMT than missense mutations. Although in each case all patients needed technical assistance with a cane or a crutch for walking after age 30, the course of the disease was more rapid in patients with homozygous truncating mutations: almost all patients over 30 (93%) were wheelchair-bound *versus *50% of patients harboring missense mutations (Table 2).

**Table 2:** Walking ability

	Walking disability			Total
	Independent	Technical assistance	Weelchair-bound	
**Recessive forms ≤ 30 y**	23 (32.9%)	28 (40%)	19 (27.1%)	70
**> 30 y**	0	6 (24%)	19 (76%)	25
*missense/missense ≤ 30 y*	*16 (45.7%)*	*10 (28.6%)*	*9 (25.7%)*	*35*
*> 30 y*	*0*	*5 (50%)*	*5 (50%)*	*10*
*truncating/truncating ≤ 30 y*	*6 (22.2%)*	*13 (48.2%)*	*8 (29.6%)*	*27*
*> 30 y*	*0*	*1 (6.7%)*	*14 (93.3%)*	*15*
*missense/truncating ≤ 30 y*	*1 (12.5%)*	*5 (62.5%)*	*2 (25%)*	*8*
*> 30 y*	*0*	*0*	*0*	*0*
**Dominant forms ≤ 30 y**	8 (73%)	3 (27%)	0	11
**> 30 y**	28 (78%)	5 (14%)	3 (8%)	36

Genotype-phenotype correlation analysis using *GDAP1 *locus-specific database data evaluated walking ability according to the mode of inheritance and the predicted effect on the protein in the recessive forms of CMT. Values represent the number of patients for each situation; the corresponding percentages are shown in brackets. y: years.

A greater number of cases of CMT disease involving *GDAP1 *mutations, along with detailed clinical, electrophysiological and pathological data, will be required to determine the relationship between the genotype and phenotype of the different forms of the disease. This is precisely the aim of our database.

### Electrophysiological data

Major axonal loss reduces the motor median nerve conduction velocity (MNCV) so that the cut-off value of MNCV should be adjusted according to the compound motor action potential (CMAP), such that the MNCV is 32 m/s when the CMAP is 4 mV, as proposed for acquired demyelinating neuropathies [25]. The demyelinating forms of CMT may thus be defined by an MNCV slower than 30 m/s, the axonal forms by an MNCV above 40 m/s, and the intermediate forms by an MNCV between 30-40 m/s [26]. We have therefore included these electrophysiological categories in our database in order to characterize the neuropathies associated with *GDAP1 *mutations. To determine the type of neuropathy, we need to report not only the MNCV but also the CMAP reflecting the axonal loss. *GDAP1 *mutations have been described as responsible for CMT4A, the demyelinating form; AR-CMT2 and CMT2K, the axonal forms; and CMTRIA, the intermediate form of CMT.

Our database contains the electrophysiological parameters for all the CMT patients reported, with the exception of 48 patients for whom the data were insufficient. Thus, our analysis concerns 131 patients among whom 30 (23%) had no recordable median nerve parameters suggesting severe axonal loss (Table 3). The majority of patients are reported to have MNCVs greater than 40 m/s, corresponding to the axonal forms of CMT and only 9 patients (7%) with MNCVs less than 30 m/s, corresponding to the demyelinating forms of CMT. However, all MNCVs less than 30 m/s (100%) were associated with major reductions of amplitude, with values less than 0.5 mV. These electrophysiological findings underscore the presence of severe axonal neuropathies with secondary demyelination in CMT associated with *GDAP1 *mutations.

**Table 3:** Analysis of electrophysiological parameters using *GDAP1 *locus-specific database data

Amplitude (mV)	Conduction Velocity (m/s)				Total
	MNCV > 40	MNCV 31-40	MNCV ≤ 30	Not recordable	
**CMAP > 6**	35 (45%)	2 (15.4%)	0	/	37
**CMAP 1.1-6**	30 (39%)	5 (23.1%)	0	/	35
**CMAP 0.5-1**	6 (8%)	3 (23.1%)	0	/	9
**CMAP < 0.5**	6 (8%)	5 (38.5%)	9 (100%)	/	20
**Not recordable**	/	/	/	30	30
**Total**	77	15	9	30	131

Values represent the number of patients for each situation; the corresponding percentages are mentioned in brackets. MNCV: motor nerve conduction velocity; CMAP: compound motor action potential.

Indeed, in demyelinating forms of CMT disease affecting myelinic proteins, such as CMT1A [MIM:118220], CMT1B [MIM:118200] [27-29] as well as in other forms of the disease, such as CMT4 [30], the conduction velocity is lower than 30 m/s even in the early phases of myelination and is definitely established between three to five years of age. This conduction velocity is independent of the clinical severity and progression of CMT, and could therefore serve as a marker of the disease even in asymptomatic patients. In contrast, the amplitudes that were initially normal decreased progressively over time following secondary axonal loss. Moreover, histopathological analyses of nerve biopsies described in cases of CMT involving *GDAP1 *mutations have revealed a loss of large myelinated fibres, which is known to reduce the MNCV [16].

Finally, the analysis of the database presented here shows that *GDAP1 *mutations are responsible for primary axonal damage in CMT, with secondary demyelination in the more severe cases of the disease. Findings of this nature should lead to a better understanding of the pathophysiology of CMT.

## Conclusions

One of the patients with a *GDAP1 *mutation referred to our database also carried a mutation in the *MFN2 *gene [31]. Such cases of multiple gene mutations are now being brought to light more often with the rapid advance of genetic investigations. The *GDAP1 *database is part of the MITOchondrial DYNamics variation portal (mitodyn.org), aimed at specializing in genes involved in disorders of mitochondrial dynamics [32]. The mitodyn.org will therefore incorporate other genes involved in diseases affecting mitochondrial dynamics and bioenergetics. Thus, the *OPA1 *LSDB (*e*OPA1) [33], curated by our laboratory, will soon be transferred to mitodyn.org. We will then integrate *MFN2*, responsible for CMT2A2 [MIM:609260] [34], and *DNM1L *associated with encephalopathy with lactic acidosis [MIM:603850] [35]. Since the same patients may be affected by these different diseases involving mitochondrial dysfunction, the data collected should interest physicians and researchers alike. Eventually, we hope to be able to refine genotype-phenotype correlations in diseases involving mitochondrial dynamics by comparing and cross-checking the clinical, electrophysiological and biochemical data recorded in the mitodyn.org databases.

## Competing interests

The authors declare that they have no competing interests.

## Authors' contributions

JC and MF initiated the project, created the database, monitored data collection and drafted the manuscript. AC participated in writing the article and the analysis of results. DB, CV, VP, PR and MF supervised the project and revised the manuscript. All authors have given final approval of the version to be published.
